# Brain Perfusion MRI Findings in Patients with Behcet's Disease

**DOI:** 10.1100/2012/261502

**Published:** 2012-04-30

**Authors:** Alpay Alkan, Asli Goktan, Yelda Karincaoglu, Suat Kamisli, Metin Dogan, Namik Oztanir, Nergiz Turan, Ercan Kocakoc

**Affiliations:** ^1^Department of Radiology, School of Medicine, Bezmialem Vakif University, İstanbul, Turkey; ^2^Department of Radiology, Kahramanmaras State Hospital, Goksun-Kahramanmaras, Turkey; ^3^Department of Dermatology, School of Medicine Inonu University, Malatya, Turkey; ^4^Department of Neurology, School of Medicine Inonu University, Malatya, Turkey; ^5^Department of Radiology, School of Medicine Inonu University, Malatya, Turkey; ^6^Department of Brain Surgery, School of Medicine Inonu University, Malatya, Turkey

## Abstract

*Objective*. To search brain perfusion MRI (pMRI) changes in Behcet's disease (BD) with or without neurological involvement. *Materials and Method*. The pMRI were performed in 34 patients with BD and 16 healthy controls. Based on neurologic examination and post-contrast MRI, 12 patients were classified as Neuro-Behcet (group 1, NBD) and 22 patients as BD without neurological involvement (group 2). Mean transit time (MTT), time to peak (TTP), relative cerebral blood volume (rCBV), and relative cerebral blood flow (rCBF) were obtained and compared to those of healthy control group (group 3). *Results*. There was a significant difference in the MTT and rCBF within the pons and parietal cortex in groups 1 and 2. rCBV increased in cerebral pedicle in group 1 compared with groups 2 and 3. In the temporal lobe white matter, prolonged MTT and decreased rCBF were found in groups 1 and 2. In the corpus striatum, internal capsule, and periventricular white matter, rCBF increased in group 1 compared with group 3 and decreased in groups 1 and 2. *Conclusion*. Brain pMRI is a very sensitive method to detect brain involvement in patients with BD and aids the clinical diagnosis of NBD, especially in patients with negative MRI findings.

## 1. Introduction

Behcet's disease (BD) was first recognized as a clinical entity by the Turkish dermatologist Hulusi Behcet. It is a multisystemic, recurrent, inflammatory disorder and affects the central nervous system (CNS) [1]. Clinical manifestations related to CNS involvement are extremely variable and are not always easily recognized [2].

Brain involvement may be caused by either primary parenchymal lesions or vascular damage. Signs or symptoms attributable to CNS involvement have been reported in 10%–50% of BD [3, 4]. MRI findings of Neuro-Behcet's disease (NBD) are nonspecific. Small irregular hyperintensities can be observed on the FLAIR and T2-weighted images. The most commonly affected region is the mesodiencephalic junction, followed by the pontobulbar region [5, 6]. Hypothalamic region, basal ganglia, cerebral hemispheres, cerebellum, and spinal cord were rarely involved [5]. The exact pathogenesis of the disease is still unknown; however; that is triggered by exogenous factors, with genetic components that predispose to the autoimmune vasculitis has been suggested [7]. Behcet's disease can cause immune-mediated vasculitis affecting small and large sized vessels. The parenchymal distribution of lesions, especially in mesodiencephalic junction, supports small vessel vasculitis involving mainly venules [8, 9]. Perivascular inflammation might be important to understanding the pathogenesis of NBD [10]. Intermittent acute perivascular inflammation might contribute to the destruction of CNS parenchyma leading to loss of neuronal functions [10]. 

 Different advanced MRI techniques, including Magnetic Resonance Spectroscopy (MRS) and Diffusion Weighted Imaging (DWI), capable of detecting subtle ultrastructural changes are now employed to depict the parenchyma involvement [11–16]. But so far, brain perfusion MRI (pMRI) findings in patients with BD have not been reported. pMRI enables assessment of regional cerebral hemodynamics using a variety of methods [17]. pMRI provides a relative and/or absolute measurement of the parameters of cerebral microvascularisation: regional blood volume, mean transit time, regional blood flow. The main applications of the first pass pMRI are vascular pathologies (vasculitis, ischemic strokes, vasospasm) and tumoral pathologies. Our aim was to investigate whether brain perfusion impairment in patients with BD with or without neurological involvement by means of pMRI occurs.

## 2. Materials and Methods

34 patients (14 female, 20 male, mean age 42.67 ± 10.74) with BD diagnosed in the Department of Dermatology were included into this study. First, neurological examinations of patients were performed. According to the results of neurological examination and conventional MRI findings, patients were classified as NBD (group 1 : 12 patients) and BD (group 2 : 22 patients). 16 healthy volunteers were included as control group (group 3). The study protocol was approved by the institutional ethical committee. All subjects were fully informed and gave their written informed consent.

We routinely performed conventional MRI and pMRI as part of the diagnostic assessment of all patients with suspected neurological involvement by BD. 1.5 T MRI system (Gyroscan Intera Master, Philips) was used for this study using a standard head coil. The routine conventional MRI sequences were used to identify anatomical structures and confirm the absence of any structural or signal abnormalities. We obtained axial T1- and T2-weighted, coronal fluid-attenuated inversion recovery (FLAIR), and sagittal T1 weighted images. Contrast-enhanced axial and coronal T1-weighted images were obtained after perfusion MRI.

For pMRI, gradient echo (FFE) sequence (TE: 30, TR: 500) with dynamic perfusion examination was performed. 16 sections and 50 dynamic examinations were performed (slice thickness: 7 mm). 0.2 mmol/kg contrast agent (gadolinium) was given using a 18 G IV catheter with automatic injection pump. Slices were obtained with a 7 sec delay. Perfusion values were determined by using software. ROIs (regions of interest) were placed into the most commonly affected areas reported in the literature. Average ROI sizes were arranged as follows: pons (70–100 mm^2^), cerebral pedicle (30–50 mm^2^), temporal lobe white matter (100–120 mm^2^), thalamus (80–120 mm^2^), the corpus striatum (200–250 mm^2^), internal capsule posterior limb (30–50 mm^2^), periventricular white matter (140–170 mm^2^), parietal cortex (30–50 mm^2^), and cerebellar white matter (130–160 mm^2^). Perfusion maps and rCBV (relative cerebral blood volume), MTT (mean transit time), rCBF (relative cerebral blood flow), and TTP (time to peak) values were obtained and compared with the control group and between groups.

## 3. Statistical Analysis

SPSS 13.0 (Statistical package for social scienses) program was used for statistical analysis. Measurable variables, mean (*x*) ± standard deviation (SD), and categorical data are presented as number and percentage. Measurable variables with the Shapiro Wilk normality test showed a normal distribution (*P* > 0.05). Comparing more than two groups (group 1, group 2, and group 3), one-way analysis of variance was used. The relationship between variables with Pearson's correlation analysis and evaluation of categorical data was tested with Pearson Chi-square analysis. *P* < 0.05 was considered statistically significant.

## 4. Results

**MRI Findings:**

**Figure 1:**
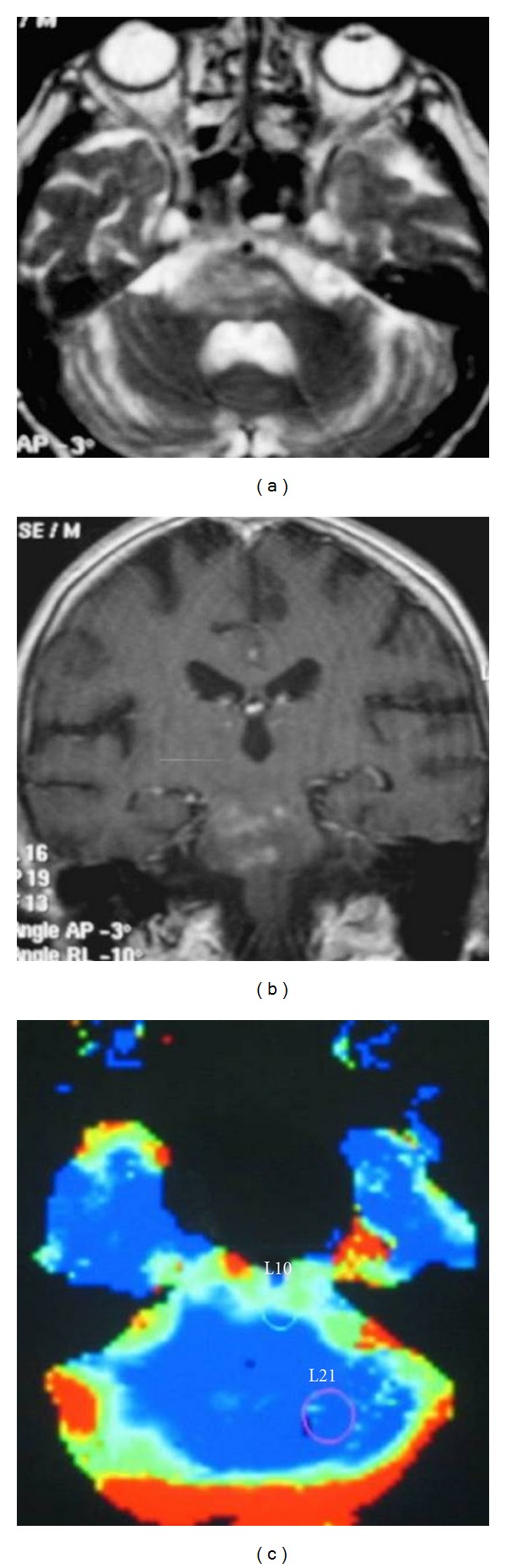
A female patient with neuro-Behcet's disease. (a) T2-weighted fast spin-echo axial MR image shows patchy hyperintensities in pons. (b) Contrast-enhanced T1-weighted echo axial MR image demonstrates nodular enhancement in pons. (c) ROIs (regions of interest) were placed into pons and cerebellar white matter in perfusion MRI.

12 patients were NBD (group 1). 22 patients (group 2) had not had neurological involvement. Pons and cerebral pedicle lesions were seen in 5 patients in MRI (Figure 1). Seven patients had involvement of the periventricular and subcortical white matter.

**pMRI Findings:**

**Figure 2:**
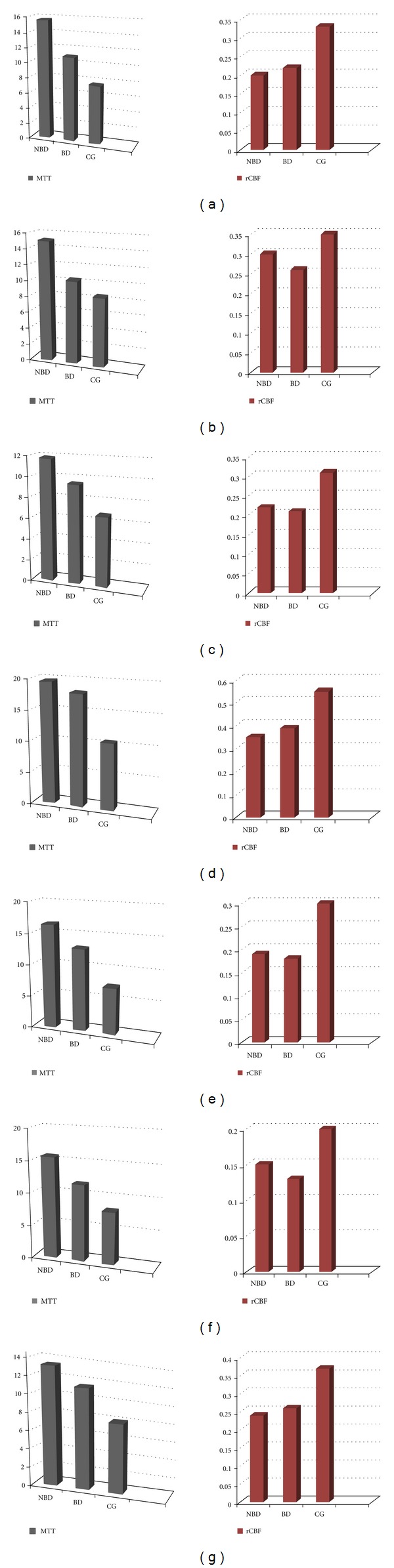
The comparative mean values of MTT and rCBF parameters between each of three groups in (a) pons (NBD: neuro-Behcet's disease (*n* : 12)), (BD: Behcet's disease (*n* : 22)), (CG: control group (*n* : 16)), (b) cerebral peduncle, (c) corpus striatum, (d) temporal lobe white matter, (e) posterior limb of internal capsule, (f) periventricular white matter, (g) parietal cortex.

- Patients with Behcet's disease (totally, *n* = 34) compared with the control group (*n* = 13) (Table 1):
  1. increased rCBV (*p*: 0,03) and prolonged MTT (*p*: 0,04) in cerebral pedicle,
  2. increased rCBV (*p*: 0,00) and prolonged TTP (*p*: 0,03) in thalamus,
  3. increased rCBV (*p*: 0,01) and prolonged MTT (*p*: 0,02) in temporal lobe white matter,
  4. prolonged MTT (*p*: 0,00) in corpus striatum,
  5. prolonged TTP (*p*: 0,03) in internal capsule posterior limb
  6. increased rCBV (*p*: 0,03), prolonged TTP (*p*: 0,02) and MTT (*p*: 0,00) in periventricular white matter,
  7. prolonged MTT (*p*: 0,01) and TTP (*p*: 0,02) in cerebellar white matter.
- neuro-Behcet's (NBD, group 1), BD (without neurologic involvement; group 2), and the control group (group 3) according to the localization of the average perfusion values of the results of statistical comparison are presented in Figure 2 and Table 2.

**Pons (Figure 2(a)):**

1. There was significant difference in MTT values between group 1-2 (*p*: 0,01), group 2-3 (*p*: 0,03), and group 1–3 (*p*: 0,00). MTT values in patients with Neuro-Behcet's (group 1) was prolonged compared to group 2 and 3. Also, MTT values in group 2 were prolonged compared to control group.
2. There was significant difference rCBF values between group 1–3 (*p*: 0,00) and group 2-3 (*p*: 0,00). rCBF values in group 1 and 2 compared to group 3 decreased.

**Cerebral Pedicle (Figure 2(b)):**

1. There was significant difference in MTT values between group 1-2 (*p*: 0,02) and group 1–3 (*p*: 0,00). MTT values in group 1 were prolonged compared to groups 2 and 3.
2. There was significant difference in rCBV values between group 1-2 (*p*: 0,02) and group 1–3 (*p*: 0,03). rCBV values in group 1 were prolonged.

**Corpus Striatum (Figure 2(c)):**

1. MTT values in group 1 compared to groups 3 were prolonged (*p*: 0,01).
2. rCBF values between groups 1 and 3 (*p*: 0,05) and groups 2 and 3 (*p*: 0,02) showed significant difference. rCBF values of group 1 and 2 values were decreased.

**Temporal Lobe White Matter (Figure 2(d)):**

1. MTT values between groups 1 and 3 (*p*: 0,01) and groups 2 and 3 (*p*: 0,01) showed significant difference. MTT values in group 1 and 2 were prolonged compared with group 3.
2. rCBF values in groups 1 and 2 compared with group 3 (*p*: 0,00, and *p*: 0,01) showed significant difference. rCBF values in group 1 and 2 were decreased.

**Internal Capsule Posterior Limb (Figure 2(e)):**

1. MTT values in group 1 also had a significant increase compared with group 3 (*p*: 0,01).
2. rCBF values in group 1 and 2 compared with group 3 (*p*: 0,00, and *p*: 0,00) were decreased.

**Periventricular White Matter (Figure 2(f)):**

1. MTT values in group 1 compared with group 3 were significantly prolonged (*p*: 0,00).
2. rCBF values in groups 1 and 2 compared with group 3 (*p*: 0,04, and *p*: 0,00) were decreased.

**Parietal Cortex (Figure 2(g)):**

1. MTT values in group 1 compared with groups 2 and 3 were prolonged (*p*: 0,00 and *p*: 0,02).
2. rCBF values in groups 1 and 2 compared with group 3 (*p*: 0,00, *p*: 0,00) were decreased.

**Cerebellar White Matter:**

1. rCBF values in group 1 and 2 compared with group 3 (*p*: 0,00, *p*: 0,00) were decreased.

## 5. Discussion

Behcet's disease is characterized clinically by the presence of a diagnostic triad of oral and genital aphthous ulcers, meningoencephalitis, and relapsing uveitis [1, 18]. The disease is usually seen in the third decade and is more common in men [19]. The disease prevalence is higher in countries on the Silk Road [7]. Familial aggregation of the disease has been reported mainly in Turkey and Japan.

Clinical and imaging data suggest that the clinical variation is also seen with the neurological involvement. BD is a systemic vasculitis of unknown origin with neurological involvement. The etiological factors remain obscure, but viral agents, immunological factors, genetic causes, bacterial factors, and fibrinolytic defects have been implicated [6, 20]. NBD is classified in two major forms: (1) focal and multifocal parenchymal involvement associated with inflammatory disease of the small veins (80%), (2) better prognosis and a more limited with symptoms of cerebral venous sinus thrombosis (20%). One is attributable to small venous inflammatory disease with focal or multifocal CNS parenchymal involvement and is seen in the majority of patients [5]. Several studies reported neuropathologic findings in NBD. It causes inflammatory changes in the meninges and brain parenchyma with perivascular lymphocytic infiltrates involving veins, venules, capillaries, and, less frequently, arteries. As the lesions become more chronic, gliosis, atrophy, and thickening and fibrosis of the meninges may ensue [4, 6, 7, 20].

Neuroimaging studies have shown that lesions are generally located within the brainstem, occasionally with extension to the diencephalon, or less commonly within the periventricular and subcortical white matter. The most commonly affected region is the mesodiencephalic junction, followed by the pontobulbar region [5, 6, 21]. Most patients who do have mesodiencephalic junction lesions also show an upward extension involving the diencephalic structures or a downward extension involving the pontobulbar region [5, 6, 20]. MRI is currently the most sensitive and prevalent tool for detecting CNS lesions. White matter lesions are usually multiple small foci of high intensity of T2-weighted images [21]. They are usually extensive, confluent, and distributed over the white matter without predilection for the periventricular regions [21]. In our study, we showed hyperintense lesions in pons and cerebral pedicles in 5 patients, in periventricular and subcortical white matter in 7 patients on T2 and FLAIR images. Hemispheric white matter lesions in patients with NB are not common. Subcortical lesions are more frequent. Subcortical lesions were found in 4 patients, and periventricular white matter lesions were demonstrated in 3 patients. 

pMRI is currently used to investigate the brain physiology in healthy patients and in those with various brain diseases. pMRI can demonstrate the altered physiologic state of the cerebral vasculature immediately after an occlusive or partially occlusive event. This physiologic state may be characterized by parameters of tissue perfusion such as relative cerebral blood volume (rCBV), relative cerebral blood flow (rCBF), and mean transit time (MTT) [22]. It can be used in brain vascular diseases (vasculitis, angiopathy, ischemic stroke), tumoral diseases (to evaluate neoangiogenesis and tumoral vascularisation), and infectious or inflammatory diseases (hypervascularisation).

pMRI seems to be very sensitive in disclosing brain abnormalities in patients with BD and signs or symptoms of CNS involvement, even with negative findings on brain MRI. Normal MRI findings cannot exclude a previous neurological attack in a patient with BD [23]. Akman-Demir et al. [23] reported 22 patients with neurological involvement without preceding neurological attacks in their study. Approximately half of the patients were normal MRI findings. However, the majority of the patients with normal MRI had silent neurological involvement [23]. Although MRI findings are normal, perfusion SPECT is sensitive about the results of brain involvement in Behcet's patients with neurological symptoms. Vignole et al. [2] studied perfusion SPECT in 7 patients with BD. Hypoperfusion in right thalamus, caudate nucleus, and left frontolateral region was detected in a patient with headache. At the beginning of the left caudate nucleus and left thalamus and in the temporal lobe in 3 patients was found hypoperfusion. In our study, prolonged MTT and decreased rCBF values were detected in corpus striatum in patients with NB. pMRI findings obtained from the corpus striatum in patients with NB indicate hypoperfusion. Otherwise, decreased rCBF values in patients with Behcet's without neurological involvement also show hypoperfusion and subclinic dysfunction in corpus striatum. 

Eight patients of BD with neurological symptoms were studied using perfusion SPECT, which disclosed areas of hypoperfusion localized in the deep basal ganglia or in the frontal and temporal cortex. MRI was normal in five of them [24]. In another similar study performed in 10 adults with neuropsychiatric manifestations related to BD [25], whose MRI were normal, SPECT demonstrated hypoperfusion areas in the brains of all patients, with the parietal and temporal lobes being the most commonly involved regions. These perfusion SPECT findings, consistent with multiple hypoperfusion areas that correlate with decreased metabolic demand, are not specific for NBD, but they may be interpreted as being indicative of early functional changes in the brains in this patient population [25].

Vignole et al., in same study [2], detected hypoperfusion in right mesiotemporal and laterotemporal cortex in a patient. Prolonged MTT and decreased rCBF values were found in temporal lobe white matter in patients with NB and BD without neurological involvement in our study. These findings confirm hypoperfusion in temporal lobe in white matter. MTT values obtained from parietal cortex in patients with NB compared with Behcet's and controls were prolonged. rCBF values in patients with NBD were decreased compared with controls. García-Burillo et al. [26] studied HMPAO brain perfusion SPECT in BD with neuropsychiatric symptoms. In patients without neurological symptoms (without MRI findings), SPECT abnormalities incompatible with the high proportion of primary indicate that blood flow deficits or focal metabolic disorder that is probably premature indicates the involvement of subclinical CNS [26]. In our study, in patients without neurological symptoms were detected prolonged MTT and decreased rCBF values in pons and temporal lobe white matter. In addition, rCBF values of corpus striatum, internal capsule, PVWM, parietal cortex, and cerebellum were decreased. Prolonged MTT and decreased rCBF values without MRI involvement suggest that subclinical dysfunction. 

Kao et al. found hypoperfusion in gray matter in all cases of 13 patients with NBD in SPECT study [27]. White matter lesions in MRI were seen in only 4 cases. In our study, MTT values obtained from parietal cortex in patients with NBD were prolonged compared to group 2 and control. In addition, rCBF values of patients with NBD and without neurological symptoms were decreased. In patients with BD, parietal cortex lesions on MRI were not detected. Decreased rCBF values in gray matter suggesting that more easily determination of metabolic and functional changes in the brain.

## 6. Conclusion

Brain perfusion MRI is a very sensitive method to detect brain involvement in patients with BD and may support the clinical diagnosis of NBD, especially in patients with negative MRI findings. We think pMRI has great potential because it provides direct information related to regional hypoperfusion caused by small vasculitic lesions. It may act as complementary modality to increase the detection rate of the affected location in patients with NBD. pMRI results in patients with BD with/or without neurological involvement indicate hypoperfusion in different regions of the brain. Hypoperfusion findings in patients with NBD were much more prominent. Detection of signs of hypoperfusion in different brain regions by pMRI in BD patients supports the idea that subclinical vascular and neuronal dysfunction develops.

## Figures and Tables

**Table 1:** Perfusion parameter values of all Behcet's patients and control group.

Locations	Total patients with Behcet's disease (*n* = 34)				Control group (*n* = 16)			
	rCBV *x* ± SD	MTT *x* ± SD	rCBF *x* ± SD	TTP *x* ± SD	rCBV *x* ± SD	MTT *x* ± SD	rCBF *x* ± SD	TTP *x* ± SD
Pons	2,54 ± 1,24	12,64 ± 5,83	0,21 ± 0,09	41,23 ± 8,08	2,40 ± 0,76	7,71 ± 1,99	0,33 ± 0,14	39,41 ± 5,55
Cerebral pedicle	3,24 ± 2,20	12,08 ± 6,52	0,27 ± 0,14	40,60 ± 7,89	2,77 ± 1,12	8,64 ± 3,24	0,35 ± 0,15	39,88 ± 5,36
Thalamus	2,86 ± 1,66	13,77 ± 14,04	0,26 ± 0,13	41,09 ± 7,75	2,40 ± 0,84	7,10 ± 1,67	0,36 ± 0,17	38,26 ± 4,80
Temporal lobe white matter	7,28 ± 5,46	18,52 ± 9,70	0,38 ± 0,15	42,64 ± 8,54	5,52 ± 2,32	10,71 ± 5,05	0,55 ± 0,22	39,16 ± 5,51
Corpus striatum	2,09 ± 1,22	10,31 ± 5,84	0,22 ± 0,12	40,56 ± 10,63	2,11 ± 0,83	6,75 ± 1,60	0,31 ± 0,12	38,16 ± 4,66
Internal capsule posterior limb	2,50 ± 1,76	14,32 ± 10,08	0,18 ± 0,08	40,32 ± 10,54	2,01 ± 0,91	7,90 ± 5,45	0,30 ± 0,11	37,93 ± 5,06
Periventricular white matter	1,93 ± 1,51	13,15 ± 7,13	0,14 ± 0,07	41,72 ± 10,02	1,71 ± 0,74	8,16 ± 3,03	0,20 ± 0,06	40,06 ± 5,04
Parietal cortex	2,77 ± 1,22	12,15 ± 5,36	0,25 ± 0,10	40,48 ± 8,56	2,76 ± 1,09	7,80 ± 3,18	0,37 ± 0,14	39,52 ± 5,12
Cerebellum	2,51 ± 1,45	12,97 ± 8,93	0,21 ± 0,09	41,57 ± 11,04	2,43 ± 0,83	7,71 ± 2,86	0,32 ± 0,11	39,4 ± 4,99

**Table 2:** Statistically significant differences in perfusion parameters are presented.

Locations		Group 1 (*n* : 12)	Group 2 (*n* : 22)	Group 3 (*n* : 16)
Pons	MTT	15,56 ± 7,34^*α*,*¥*^	11,05 ± 4,20^*α*,*β*^	7,71 ± 1,99^*β*,*¥*^
	rCBF	0,20 ± 0,11^*¥*^	0,22 ± 0,08^*β*^	0,33 ± 0,14^*β*,*¥*^
Cerebral pedicle	rCBV	4,26 ± 2,82^*α*,*¥*^	2,68 ± 1,58^*α*^	2,77 ± 1,12^*¥*^
	MTT	15,10 ± 7,97^*α*,*¥*^	10,43 ± 5,06^*α*^	8,64 ± 3,24^*¥*^
Temporal lobe white matter	MTT	19,54 ± 10,90^*β*,*¥*^	17,97 ± 9,20^*β*^	10,71 ± 5,05^*β*,*¥*^
	rCBF	0,35 ± 0,15^*β*,*¥*^	0,39 ± 0,15^*β*^	0,55 ± 0,22^*β*,*¥*^
Corpus striatum	MTT	11,73 ± 4,16^*¥*^	9,53 ± 6,54	6,75 ± 1,60^*¥*^
	rCBF	0,22 ± 0,14^*¥*^	0,21 ± 0,11^*β*^	0,31 ± 0,12^*β*,*¥*^
Internal capsul posterior limb	MTT	16,49 ± 14,22^*¥*^	13,14 ± 7,04	7,90 ± 5,45^*¥*^
	rCBF	0,19 ± 0,09^*¥*^	0,18 ± 0,07^*β*^	0,30 ± 0,11^*β*,*¥*^
Periventricular white matter	MTT	15,65 ± 5,37^*¥*^	11,79 ± 7,70	8,16 ± 3,03^*¥*^
	rCBF	0,15 ± 0,09^*¥*^	0,13 ± 0,05^*β*^	0,20 ± 0,06^*β*,*¥*^
Parietal cortex	MTT	13,56 ± 6,73^*¥*^	11,38 ± 4,43^*β*^	7,80 ± 3,18^*β*,*¥*^
	rCBF	0,24 ± 0,07^*¥*^	0,26 ± 0,11^*β*^	0,37 ± 0,14^*β*,*¥*^
Cerebellum	rCBF	0,18 ± 0,09^*¥*^	0,23 ± 0,09^*β*^	0,32 ± 0,11^*β*,*¥*^

(a) Group 1 compared with group 2^*α*^

(b) Group 2 compared with group 3^*β*^

(c) Group 1 compared with group 3^¥^.

## References

[1] Krespi Y, Akman-Demir G, Poyraz M (2001). Cerebral vasculitis and ischaemic stroke in Behçet’s disease: report of one case and review of the literature. *European Journal of Neurology*.

[2] Vignola S, Nobili F, Picco P (2001). Brain perfusion SPECT in juvenile neuro-Behçet’s disease. *Journal of Nuclear Medicine*.

[3] Arai Y, Kohno S, Takahashi Y, Miyajima Y, Tsutusi Y (2006). Autopsy case of neuro-Behçet’s disease with multifocal neutrophilic perivascular inflammation. *Neuropathology*.

[4] Akman-Demir G, Serdaroglu P, Tasçi B (1999). Clinical patterns of neurological involvement in Behcet’s disease: evaluation of 200 patients. *Brain*.

[5] Siva A, Altintas A, Saip S (2004). Behçet’s syndrome and the nervous system. *Current Opinion in Neurology*.

[6] Koçer N, Islak C, Siva A (1999). CNS involvement in Neuro-Behcet syndrome: an MR study. *American Journal of Neuroradiology*.

[7] Haghighi AB, Sharifzad HR, Matin S, Rezaee S (2007). The pathological presentations of neuro-Behçet disease: a case report and review of the literature. *The Neurologist*.

[8] Moritani T, Hiwatashi A, Shrier DA, Wang HZ, Numaguchi Y, Westesson PLA (2004). CNS vasculitis and vasculopathy: efficacy and usefulness of diffusion-weighted echoplanar MR imaging. *Clinical Imaging*.

[9] Scully SE, Stebner FC, Yoest SM (2004). Magnetic resonance spectroscopic findings in neuro-behçet disease. *The Neurologist*.

[10] Arai Y, Kohno S, Takahashi Y, Miyajima Y, Tsutusi Y (2006). Autopsy case of neuro-Behçet’s disease with multifocal neutrophilic perivascular inflammation. *Neuropathology*.

[11] Baysal T, Dogan M, Karlidag R (2005). Diffusion-weighted imaging in chronic Behçet patients with and without neurological findings. *Neuroradiology*.

[12] Kang DW, Chu K, Cho JY (2001). Diffusion weighted magnetic resonance imaging in neuro-Behçet's disease. *Journal of Neurology Neurosurgery and Psychiatry*.

[13] Kunimatsu A, Abe O, Aoki S (2003). Neuro-Behçt’s disease: analysis of apparent diffusion coefficients. *Neuroradiology*.

[14] Sener RN (2003). Neuro-Behcet’s disease: diffusion mr imaging and proton MR spectroscopy. *American Journal of Neuroradiology*.

[15] Ciliz D, Sakman B, Fettahoglu EF, Yuksel E (2001). Nöro-Behçet hastalığında kranyal MRG bulguları. *Tanısal ve Girişimsel Radyoloji*.

[16] Koné-Paut I, Chabrol B, Riss JM, Mancini J, Raybaud C, Garnier JM (1997). Neurologic onset of Behcet’s disease: a diagnostic enigma in childhood. *Journal of Child Neurology*.

[17] Luypaert R, Boujraf S, Sourbron S, Osteaux M (2001). Diffusion and perfusion MRI: basic physics. *European Journal of Radiology*.

[18] Behçet H (1937). Uber rezidivierende aphthose, durch ein virus verursachte geschwure am mund, am auge und an den genitalien. *Dermatologische Wochenschrift*.

[19] Tali ET, Atilla S, Keskin T, Simonson T, Işik S, Yuh WTC (1997). MRI in neuro-Behcet’s disease. *Neuroradiology*.

[20] Hiwatashi A, Garber T, Moritani T, Kinoshita T, Westesson PL (2003). Diffusion-weighted MR imaging of neuro-Behçet’s disease: a case report. *Neuroradiology*.

[21] Moritani T, Hiwatashi A, Shrier DA, Wang HZ, Numaguchi Y, Westesson PLA (2004). CNS vasculitis and vasculopathy: efficacy and usefulness of diffusion-weighted echoplanar MR imaging. *Clinical Imaging*.

[22] Schaefer PW, Ozsunar Y, He J (2003). Assessing tissue viability with MR diffusion and perfusion imaging. *American Journal of Neuroradiology*.

[23] Akman-Demir G, Bahar S, Coban O, Tasci B, Serdaroglu P (2003). Cranial MRI in Behçet’s disease: 134 examinations of 98 patients. *Neuroradiology*.

[24] Nobili F, Cutolo M, Sulli A, Vitali P, Vignola S, Rodriguez G (2002). Brain functional involvement by perfusion SPECT in systemic sclerosis and Behçet’s disease. *Annals of the New York Academy of Sciences*.

[25] Huang WS, Chiu PY, Kao A, Tsai CH, Lee CC (2002). Decreased cerebral blood flow in neuro-Behcet’s syndrome patients with neuropsychiatric manifestations and normal magnetic resonance imaging—a preliminary report. *Journal of Neuroimaging*.

[26] García-Burillo A, Castell J, Fraile M (1998). Technetium-99m-HMPAO brain SPECT in Behcet’s disease. *Journal of Nuclear Medicine*.

[27] Kao CH, Lan JL, Changlai SP, Chieng PU (1998). Technetium-99m-HMPAO SPECT and MRI of brain in patients with Neuro- Behcet’s syndrome. *Journal of Nuclear Medicine*.

